# Prediction models for postoperative delirium in elderly patients with machine-learning algorithms and SHapley Additive exPlanations

**DOI:** 10.1038/s41398-024-02762-w

**Published:** 2024-01-25

**Authors:** Yuxiang Song, Di Zhang, Qian Wang, Yuqing Liu, Kunsha Chen, Jingjia Sun, Likai Shi, Baowei Li, Xiaodong Yang, Weidong Mi, Jiangbei Cao

**Affiliations:** 1 Department of Anesthesiology, The First Medical Center of PLA General Hospital, Beijing, China; 2 Institute of Computing Technology, Chinese Academy of Sciences, Beijing, China; 3 National Clinical Research Center for Geriatric Diseases, People’s Liberation Army General Hospital, 100853 Beijing, China

**Keywords:** Human behaviour, Diseases

## Abstract

Postoperative delirium (POD) is a common and severe complication in elderly patients with hip fractures. Identifying high-risk patients with POD can help improve the outcome of patients with hip fractures. We conducted a retrospective study on elderly patients (≥65 years of age) who underwent orthopedic surgery with hip fracture between January 2014 and August 2019. Conventional logistic regression and five machine-learning algorithms were used to construct prediction models of POD. A nomogram for POD prediction was built with the logistic regression method. The area under the receiver operating characteristic curve (AUC-ROC), accuracy, sensitivity, and precision were calculated to evaluate different models. Feature importance of individuals was interpreted using Shapley Additive Explanations (SHAP). About 797 patients were enrolled in the study, with the incidence of POD at 9.28% (74/797). The age, renal insufficiency, chronic obstructive pulmonary disease (COPD), use of antipsychotics, lactate dehydrogenase (LDH), and C-reactive protein are used to build a nomogram for POD with an AUC of 0.71. The AUCs of five machine-learning models are 0.81 (Random Forest), 0.80 (GBM), 0.68 (AdaBoost), 0.77 (XGBoost), and 0.70 (SVM). The sensitivities of the six models range from 68.8% (logistic regression and SVM) to 91.9% (Random Forest). The precisions of the six machine-learning models range from 18.3% (logistic regression) to 67.8% (SVM). Six prediction models of POD in patients with hip fractures were constructed using logistic regression and five machine-learning algorithms. The application of machine-learning algorithms could provide convenient POD risk stratification to benefit elderly hip fracture patients.

## Introduction

Hip fracture is a common type of fracture in elderly patients. By 2050, it is estimated that more than 50% of osteoporotic fractures will be hip fractures in Asia [1]. As life expectancy increases, more elderly patients choose surgery to treat hip fractures for a better prognosis. Postoperative delirium (POD) is a common and severe complication in patients with hip fractures [2–4]. It is common for POD to occur 2–7 days after surgery. POD is associated with loss of independence, increased morbidity and mortality, institutionalization, and a prolonged hospital stay with higher healthcare costs [3, 5]. Researchers have found that multifactor prevention and treatment can benefit one-third of delirium cases [6]. By identifying high-risk patients, clinicians can improve the outcomes of patients with hip fractures through timely intervention.

In various clinical domains, machine-learning methods have proven helpful in predicting events of interest [7–10]. Some studies have developed POD prediction models in hip fracture patients with conventional logistic regression methods [11–15], but few have proposed prediction models with machine learning. Furthermore, the results of these studies were not entirely satisfied for the areas under the receiver operating curve (AUCs) of 0.779-0.79 [16, 17]. More attempts should be presented for better predicting POD in hip fracture patients using machine-learning methods.

Thus, we try to develop a prediction model of POD with conventional logistic regression and machine-learning algorithms to support clinical decision-making.

## Materials and methods

### Study design and patients

Our study was retrospective. From January 2014 to April 2019, a cohort of Chinese PLA General Hospital patients who underwent hip fracture surgery was analyzed in this study. The inclusion criteria were: (1) age ≥65 years; (2) undergoing surgery for hip fracture with anesthesia. The exclusion criteria were: (1) undergoing secondary surgery for hip fracture；(2) hip fractures caused by tumors.

### Ethics statements

According to the Declaration of Helsinki, the study was approved by the Ethics Committee Board of the First Medical Center of the Chinese PLA General Hospital (Number: S2019-311-03). All data were anonymized before analysis, and patient consent was waived due to the retrospective study design.

### Data collection

The dataset of hip fractures was established from the medical record system. We collected preoperative and intraoperative parameters. The basic characteristics of patients included age, sex, body mass index (BMI), smoking, alcohol, history of hypertension, diabetes, cardiovascular diseases (CHD), chronic obstructive pulmonary disease (COPD), renal insufficiency, cerebrovascular disease, depression, and anxiety. Before surgery, the prescribed medication included anticholinergic drugs, non-steroidal anti-inflammatory drugs (NSAIDs), benzodiazepines, opioids, and antipsychotic drugs were recorded. The laboratory test results of the last time before surgery were collected: the complete blood cell count (CBC), Arterial Blood Gas (ABG), Clotting factors, and Comprehensive Metabolic Panel (CMP). Some intraoperative data were recorded: American Society of Anesthesiologists (ASA) physical status classification, the type of hip fracture, the type of surgery and anesthesia, duration of surgery and anesthesia, urine, blood loss, use of dexmedetomidine and droperidol, fluid management (crystalloid and colloid), blood transfusion, use of glucocorticoids (dexamethasone and methylprednisolone), dexmedetomidine, droperidol, vasoactive drugs, preoperative hospital stay, duration of systolic blood pressure (SBP) >=140 mmHg, and mean arterial pressure (MAP) <=60 mmHg.

### Definitions of POD

The incidence of POD within consecutive 7 days postoperatively was recorded. First, the patients with characteristic words of delirium documented in the postoperative medical records were captured by the computer. All the characteristic words of delirium were chosen according to the Confusion Assessment Method (CAM) scale [18, 19]. Second, the patients using drugs for delirium postoperatively were also added. Third, the patients with preoperative medical records containing the words of delirium and the drug for delirium were excluded. At last, all the patients preliminarily diagnosed by a computer were rechecked by neurologists using the Diagnostic and Statistical Manual of Mental Disorders, Fourth Edition (DSM-IV) criteria [20].

### Model building strategy

A predictive model using logistic regression was developed. The training and validation datasets were randomly divided by 3:1. The variables in the model were selected using forward and backward stepwise methods. The nomogram of the prediction model was then established. Patients from the validation dataset were used to evaluate the prediction model. The area under the receiver operating characteristic curve (AUC) was calculated to assess the prediction model’s discrimination ability. Hosmer–Lemeshow goodness-of-fit testing was used to assess the model’s calibration. For each threshold probability, a decision curve analysis (DCA) revealed the net benefits [21].

We developed five different machine-learning models with different algorithms: random forest (RF), Support Vector Machines (SVM), adaptive boosting with classification trees (AdaBoost), extreme gradient boosting with classification trees (XGBoost), and gradient boosting machine (GBM). The k-fold cross-validation (*k* = 5) was used for training since it is simple to understand and generally results in a less biased or optimistic estimate of the model skill than other methods [22]. An over-sampling method was used for the nonequilibrium dataset (many negative and very few positive patients) to improve machine-learning models’ performances. We used an improved over-sampling algorithm named borderline SMOTE in constructing our machine-learning models. The algorithm uses only minority class samples on the border to synthesize new samples, thereby improving the class distribution of the samples. After using borderline SMOTE, the model performance reached its best.

The interpretability of the model was used SHapley Additive exPlanations (SHAP). Feature importance of different individuals was shown in SHAP figures.

### Statistical analysis

In this study, Student’s t-tests were used to compare normally distributed continuous variables, expressed as mean (standard deviation). A Mann–Whitney’s test compared continuous variables under non-normal distribution expressed as median and interquartile range. The *χ*^2^ test or Fisher’s exact test compares the categorical variables expressed as frequency or percentage. The significance level was set at 0.05, and all tests were two-tailed. The logistic regression model was developed with R 4.0.1 (R Foundation for Statistical Computing, Vienna, Austria). Machine-learning models were constructed with PyCharm 11.0.14.1 (JetBrains s.r.o., Prague, Czech Republic).

## Results

### Baseline characteristics of patients

From January 2014 to August 2019, 812 elderly patients (>=65 years old) underwent surgery for hip fractures at the First Medical Center of Chinese PLA General Hospital. We excluded 14 patients whose hip fractures were caused by tumors and one patient who underwent surgery for a hip fracture for the second time. At last, 797 patients were enrolled in the final analysis. The incidence of delirium was 9.28% (74/797). Males comprised 23.7% of the enrolled patients (189/797). The POD patients were older than non-POD patients (83 vs. 79, *P* < 0.001).

Tables 1 and 2 show the characteristics and perioperative variables of the 797 patients. The median age of POD patients was significantly older than non-PODs [83(76.25,87) vs. 79(73,84)]. The incidence of depression/anxiety, renal insufficiency, and COPD in POD patients was higher than in non-POD patients. The use of benzodiazepines and antipsychotics in POD patients was more common than in non-POD patients (32.4% vs. 20.1%, 17.6% vs. 2.1%). The median duration of surgery was 100 (80,120) min. Compared to non-POD patients, the POD patients had higher Troponin T, Myoglobin, Brain Natriuretic Peptide (BNP), and Creatine Kinase-MB(CK-MB) (*P* ≤ 0.001).

**Table 1:** Patient characteristics and baseline variables. Data are mean (standard deviation), *n* (%), or median (interquartile range).

Characteristics	Non-POD (*n* = 723)	POD (*n* = 74)	*P*-value
Sex (male), *n* (%)	167 (23.1)	22 (29.7)	0.257
Age, years, median (IQR)	79 (73,84)	83 (76.25,87)	<0.001
BMI, kg·m^2^, median (IQR)	22.8 (20.03,25.635)	22.53 (19.613,24.755)	0.313
Smoking, *n* (%)	55 (7.6)	8 (10.8)	0.455
Drinking, *n* (%)	39 (5.4)	4 (5.4)	>0.999
Hypertension, *n* (%)	418 (57.8)	41 (55.4)	0.783
Diabetes, *n* (%)	231 (32)	30 (40.5)	0.171
Coronary heart disease, *n* (%)	116 (16)	11 (14.9)	0.923
Cerebrovascular disease, *n* (%)	109 (15.1)	16 (21.6)	0.191
Depression/anxiety, *n* (%)			0.002
Depression	11 (1.5)	3 (4.1)	
Anxiety	0	1 (1.4)	
Renal insufficiency, *n* (%)	18 (2.5)	8 (10.8)	<0.001
COPD, *n* (%)	40 (5.5)	10 (13.5)	0.014
Type of hip fracture, *n* (%)			0.522
Femoral neck fracture	364 (50.3)	35 (47.3)	
Intertrochanteric fracture	350 (48.4)	39 (52.7)	
Others	9 (1.2)	0	
Type of surgery, *n* (%)			0.547
Closed reduction and internal fixation	355 (49.1)	35 (47.3)	
Open reduction and internal fixation	358 (49.5)	39 (52.7)	
Joint replacement surgery	10 (1.4)	0	
Duration of operation (min), median (IQR)	100 (80,120)	95 (75,118.75)	0.229
Duration of anesthesia(min), median (IQR)	160 (137,185)	160 (135,183.75)	0.504
ASA classification, *n* (%)			0.001
I	1 (0.1)	0	
II	360 (49.8)	25 (33.8)	
III	346 (47.9)	44 (59.5)	
IV	16 (2.2)	4 (5.4)	
V	0	1 (1.4)	
Type of anesthesia, *n* (%)			0.162
Spinal anesthesia	75 (10.4)	3 (4.1)	
Nerve block	136 (18.8)	15 (20.3)	
General anesthesia	146 (20.2)	11 (14.9)	
General anesthesia combined with other modalities	366 (50.6)	45 (60.8)	
Urine (mL), median (IQR)	200 (100,400)	200 (100,300)	0.107
Blood loss (mL), median (IQR)	150 (100,200)	150 (100,200)	0.998
Colloid (mL), median (IQR)	0 (0,500)	0 (0,500)	0.883
Crystal (mL), median (IQR)	1100 (700,1200)	1050 (700,1100)	0.557
Blood transfusion, *n* (%)	243 (33.6)	36 (48.6)	0.014
Intraoperative blood pressure			
Duration of SBP > = 140 mmHg(min), median (IQR)	20 (0,45)	15 (0,50)	0.995
Duration of MAP < 60 mmHg(min), median (IQR)	5 (0,10)	5 (0,10)	0.905
Preoperative hospital stay (days), median (IQR)	6 (4,8)	6 (4,8)	0.317

*BMI* body mass index, *COPD* chronic obstructive pulmonary disease, *ASA* American Society of Anesthesiologists physical status classification system, *SBP* systolic blood pressure, *MAP* mean arterial pressure.

**Table 2:** The preoperative laboratory testing and perioperative medication. Data are mean (standard deviation), *n* (%), or median (interquartile range).

Characteristics	Non-POD (*n* = 723)	POD (*n* = 74)	*P*-value
Troponin T (ug/L), median (IQR)	0.011(0.007,0.015)	0.016 (0.01,0.026)	<0.001
Hemoglobin (g/L), median (IQR)	115 (102,126)	110 (100.25,120.75)	0.161
RBC (*10^12^/L), mean (SD)	3.76 (0.55)	3.72 (0.65)	0.606
WBC (*10^9^/L), median (IQR)	6.93 (5.52,8.515)	7.2 (5.457,9.068)	0.407
Neutrophils, median (IQR)	0.71 (0.65,0.77)	0.755 (0.68,0.81)	0.003
Lymphocytes, median (IQR)	0.19 (0.14,0.25)	0.16 (0.11,0.21)	0.001
Monocytes, median (IQR)	0.07 (0.05,0.08)	0.07 (0.05,0.08)	0.437
Platelet (*10^9^/L), median (IQR)	213 (168,265)	209.5 (155,251.75)	0.523
Glucose (mmol/L), median (IQR)	5.82 (5.2,7.03)	6.225 (5.295,7.213)	0.251
Serum albumin (g/L), median (IQR)	34.6 (32.1,37.2)	33.25 (31.425,36.35)	0.057
Myoglobin quantification (ug/L), median (IQR)	43.2 (30.15,61.25)	56.665 (38.275,100.213)	<0.001
BUN (mmol/L), median (IQR)	5.54 (4.375,7.215)	6.225 (4.63,8.237)	0.026
Scr (umol/L), median (IQR)	63.8 (53.8,75.55)	64.85 (54.775,83.55)	0.135
Serum uric acid (umol/L), median (IQR)	228.1 (174.7,287.65)	214.1 (165.775,283.825)	0.471
K (mmol/L), median (IQR)	4 (3.76,4.28)	3.99 (3.8,4.272)	0.731
Na (mmol/L), median (IQR)	139.6 (137.1,141.6)	139.05 (137.425,141.95)	0.974
BNP (pg/mL), median (IQR)	259 (115.75,667.4)	602.2 (290.025,1103.5)	<0.001
Ca (mmol/L), median (IQR)	2.18 (2.1,2.25)	2.155 (2.085,2.297)	0.892
P (mmol/L), median (IQR)	1.01 (0.87,1.14)	0.95 (0.82,1.118)	0.088
Mg (mmol/L), median (IQR)	0.86 (0.81,0.91)	0.865 (0.81,0.91)	0.883
Total bilirubin (μmol/L), median (IQR)	11.5 (8.55,16.4)	12.65 (8.7,17.075)	0.31
Direct bilirubin (μmol/L), median (IQR)	3.4 (2.3,5)	4.1 (2.725,5.65)	0.03
ALT (U/L), median (IQR)	14.8 (10.75,23.1)	14.4 (11,21.375)	0.657
AST (U/L), median (IQR)	18.4 (14.6,25.3)	18.75 (14.85,27.45)	0.634
LDH (U/L), median (IQR)	189.9 (164.85,218.5)	204 (173.45,236.725)	0.015
CK-MB (U/L), median (IQR)	1.48 (1.125,2.025)	1.625 (1.405,2.348)	0.001
GGT (U/L), median (IQR)	19.1 (13.35,31.55)	20.45 (12.1,32.875)	0.722
ALP (U/L), median (IQR)	70 (57.85,85.1)	65.3 (54.35,89)	0.515
Amylase (U/L), median (IQR)	51 (38.3,66.5)	48.05 (35.4,59.175)	0.091
Lipase (U/L), median (IQR)	92.6 (65.55,129.35)	90.8 (64.725,121.275)	0.586
CRP (mg/L), median (IQR)	2.26 (0.91,4.46)	2.31 (1.182,6.09)	0.186
PaO_2_(mmHg), median (IQR)	78 (70.9,89)	74 (66,83.5)	0.008
PaCO_2_ (mmHg), median (IQR)	40.8 (37.1,44.7)	40.15 (37.1,43.775)	0.505
SPO_2_ (%), median (IQR)	95.8 (94.2,97)	94.75 (93.425,96.8)	0.015
BE (mmol/L), median (IQR)	−0.8 (−2,0.7)	−0.7 (−2.15,0.6)	0.937
TT (s), median (IQR)	15.4 (14.8,16.3)	15.7 (14.725,16.275)	0.888
APTT (s), median (IQR)	39.2 (35.45,43.6)	38.85 (35.8,42.725)	0.995
PT (s), median (IQR)	13.8 (13.3,14.4)	14.15 (13.3,14.6)	0.068
PTA (%), median (IQR)	91 (83,98)	85 (80,96.75)	0.026
INR, median (IQR)	1.06 (1.01,1.12)	1.1 (1.03,1.157)	0.026
FIB (g/L), median (IQR)	4.47 (3.855,5.24)	4.665 (3.735,5.272)	0.567
D-dimer (mg/L), median (IQR)	2.24 (1.485,3.665)	2.295 (1.402,3.3)	0.555
Preoperative medication			
Anticholinergics, *n* (%)	39 (5.4)	4 (5.4)	>0.999
Benzodiazepines, *n* (%)	145 (20.1)	24 (32.4)	0.02
NSAIDs, *n* (%)	12 (1.7)	2 (2.7)	0.379
Opioids, *n* (%)	149 (20.6)	18 (24.3)	0.55
Antipsychotics, *n* (%)	15 (2.1)	13 (17.6)	<0.001
Intraoperative medication			
Glucocorticoids, *n* (%)	224 (31)	25 (33.8)	0.716
Dexmedetomidine, *n* (%)	145 (20.1)	21 (28.4)	0.126
Droperidol, *n* (%)	81 (11.2)	11 (14.9)	0.455
Vasoactive drugs, *n* (%)	139 (19.2)	21 (28.4)	0.085

*NSAIDs* non-steroidal anti-inflammatory drugs, *RBC* red blood cell, *WBC* white blood cell, *BUN* blood urea nitrogen, *Scr* serum creatinine, *BNP* brain natriuretic peptide, *ALT* alanine aminotransferase, *AST* aspartate aminotransferase, *LDH* lactate dehydrogenase, *CK* creatine kinase, *CK-MB* creatine kinase-MB, *GGT* γ-glutamyl transferase, *ALP* alkaline phosphatase, *CRP* C-reactive protein, *PaO*_*2*_ oxygen partial pressure, *PaCO*_*2*_ partial pressure of carbon dioxide, *SPO*_*2*_ pulse oxygen saturation, *BE* base excess, *TT* thrombin time, *APTT* activated partial thromboplastin time, *PT* prothrombin time, *PTA* plasma prothrombin activity, *INR* international normalized ratio, *FIB* plasma fibrinogen.

### Development of a nomogram with logistic regression

557 patients in the training dataset were used to develop the logistic regression model. In the Supplementary File, Table S1 shows the univariate logistic regression analysis results. Variables statistically significant in the univariate analysis were included in the multivariate logistic regression analysis. Among elderly patients with hip fractures, age, renal insufficiency, antipsychotics, COPD, LDH, and CRP were independent risk factors for POD (shown in Table 3). The collinearity diagnostics were performed to multicollinearity among the risk factors. The variance inflation factors of the independent risk factors were all <2. In the univariate model, neutrophils, lymphocytes, inorganic phosphorus, myoglobin, lipase, direct bilirubin, AST, SPO_2_, PT, PTA, INR and use of intraoperative vasoactive drugs were statistically significant, but not in the multivariate model.

**Table 3:** Multivariable logistic regression model of study variables vs. POD in the training dataset.

Variables	Odds ratio (95%CI)	*P*-value
Age, years	1.079 (1.032–1.131)	0.001
Renal insufficiency, yes vs. no	4.845 (1.199–16.417)	0.016
COPD	4.518 (1.708–11.089)	0.001
Antipsychotics, yes vs. no	6.702 (2.158–19.972)	0.001
LDH (U/L)	1.009 (1.002–1.015)	0.005
CRP (%)	1.114 (1.022–1.211)	0.012

*COPD* chronic obstructive pulmonary disease, *LDH* lactate dehydrogenase, *CRP* C-reactive protein.

**Fig. 1:**
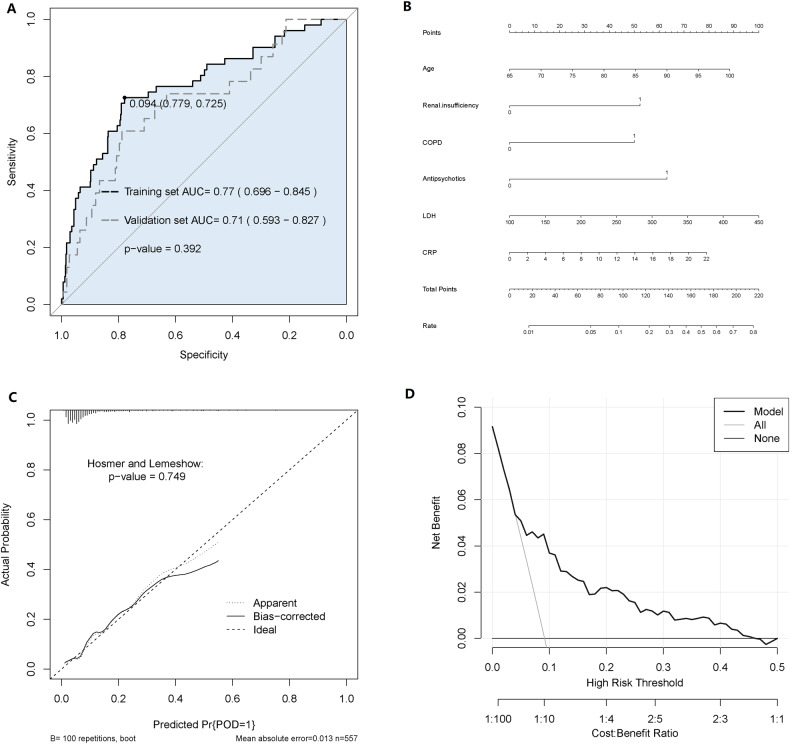
Logistic regression algorithm predicts ROC curve, nomogram, DCA curve and calibration curve of the model. **A** ROC curve of logistic regression in the training dataset and validation dataset. **B** The nomogram of the logistic regression model. This nomogram was developed with six perioperative predictors. Find each predictor’s point on the uppermost point scale and add them up. The total point projected to the bottom scale indicates the % probability of POD. **C** The calibration curve of the logistic regression model. **D** The DCA of the logistic regression model for the training dataset. DCA decision curve analysis.

The prediction model was evaluated on 240 patients in the validation dataset. The AUCs of the training dataset and the validation dataset were 0.77 (0.696–0.845) and 0.71 (0.593–0.827) (Fig. 1A). The accuracy, recall, and precision were 68.8%, 65.2%, and 18.3% in logistic regression (Table 4). The nomogram of the prediction model was developed with the six variables and their points (Fig. 1B). The calibration plot revealed good predictive accuracy between the actual and predicted probability by Hosmer–Lemeshow test (*P* = 0.749) (Fig. 1C). According to the DCA of the training dataset, except for a small range of low preferences, intervening based on the prediction model produced excellent outcomes (Fig. 1D).

**Table 4:** Comparison of the parameters of models for prediction of POD.

	AUC	Accuracy	Sensitivity (recall)	Precision	F1
RF	0.81	91.9%	95.9%	56.7%	59.6%
GBM	0.80	91.3%	76.1%	59.3%	62.7%
AdaBoost	0.68	90.6%	71.4%	62.0%	64.9%
XGBoost	0.77	91.3%	79.2%	56.3%	58.8%
SVM	0.70	68.8%	56.7%	67.8%	54.3%
LR	0.71	68.8%	65.2%	18.3%	28.5%

*AUC* area under the curve of ROC, *RF* random forest, *GBM* gradient boosting machine, *AdaBoost* adaptive boosting, *XGBoost* eXtreme gradient boosting, *SVM* support vector machine, *LR* logistic regression;

### Development of prediction models with machine-learning algorithms

All variables were preprocessed before the machine-learning models were constructed. The top variables in the normalized importance are BNP, troponin T, CRP, and CK-MB. Table S2 and Fig. S1 of the Supplementary File show the variables’ quantified importance. Moreover, the variables’ correlation was also calculated and displayed in Fig. S2 (Supplementary File).

**Fig. 2:**
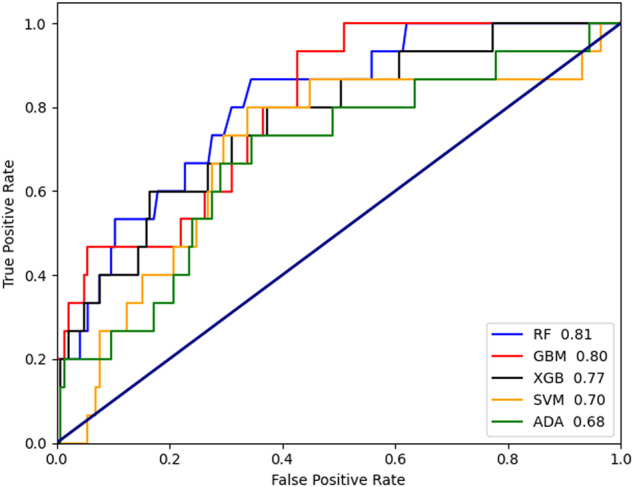
The ROCs and AUCs of POD prediction models using the various machine-learning algorithms. ROC receiver operating characteristic curve, AUC area under the curve of ROC, RF random forest, GBM gradient boosting algorithm, XGB XGBoost, SVM support vector machine, ADA AdaBoost.

The AUCs of models with different machine-learning algorithms are shown in Fig. 2. The model of RF performed best of 5 models with an AUC of 0.81. Models’ accuracy, sensitivity, precision, and F1 were calculated with a confusion matrix (Table 4). The accuracy ranged from 68.8%–91.9% in 5 models. RF performed the best sensitivity up to 95.9%. The precision of SVM was the highest (67.8%).

**Fig. 3:**
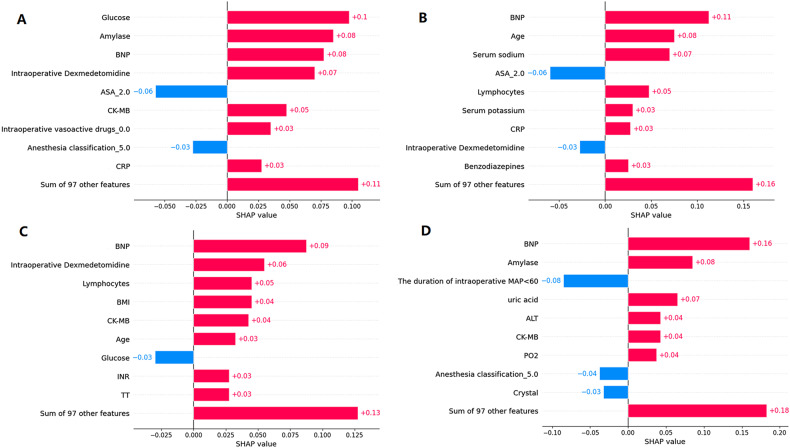
The SHAP values of the top 9 variables for four patients. **A** Patient 1. **B** Patient 2. **C** Patient 3. **D** Patient 4. SHAP Shapley additive explanations.

Model interpretation at the individual level was performed using the SHAP algorithms. We inputted the information of four different patients into the model, and the RF model provided a ranking of the importance of variables for each patient (Fig. 3A–D). Contributions of different predictors differed among individuals with different SHAP values. BNP level was the top variable in 3 patients of all 4 patients. The result was similar to the importance plots of all the models. Although causality could not be established based on the current study design, it is conceivable that individualized modification of these factors (lowering BNP and lowering amylase) may help to reduce the risk of POD.

## Discussion

Hip fractures have a devastating effect on the quality of life and function, with a high risk of death in one year. Timely surgery is the primary method of treatment for the elderly after a hip fracture [1]. However, the incidence of delirium in patients after hip arthroplasty surgeries can range from 4% to 53% [23]. It’s crucial to screen high-risk patients with preoperative and intraoperative factors as the first step toward effective management. So, one logistic regression model and five machine-learning models of POD prediction were developed in our retrospective cohort study. The AUCs of the logistic regression model were 0.77 in the training dataset and 0.71 in the validation dataset. The results were almost identical to Kim, E. M.’s risk score for POD prediction [13]. The risk score developed by Kim, E.M. for predicting postoperative delirium in patients undergoing hip arthroplasty surgery includes nine variables. However, in our logistic regression model, we only included six parameters and achieved an AUC of 0.77 in the training dataset. Similar studies that used logistic regression have also been conducted, with AUC values ranging from 0.67 to 0.79 [11, 14, 24].

With the growing application of machine-learning algorithms in medicine, some researchers have tried to develop POD prediction models of hip fractures with machine-learning algorithms. Oosterhoff et al. developed five POD prediction models using different machine-learning algorithms for hip fracture patients, with the neural network and elastic-net penalized logistic regression models performing best, achieving an AUC of 0.79 [17]. Zhao H. et al. also used four machine-learning algorithms to construct POD prediction models of hip fracture in a cohort of 245 patients, with an AUC of 0.779 [16]. In our study, we developed five different machine-learning models for predicting POD in hip fracture patients. Among these models, the random forest model achieved the best performance, with an AUC of 0.81. Interestingly, the random forest model also performed best in our previous study on POD prediction [10]. Shen J. et al. developed a risk score for predicting POD in hip fracture patients, using nine variables, and achieving an AUC of 0.833 [25]. Yang Y. et al. constructed a nomogram for POD prediction using only three variables and achieved an AUC of 0.84. Notably, these studies achieved high AUCs by including patients who had delirium before surgery. Preoperative delirium has been identified as an independent risk factor for POD in previous studies [26]. However, our study excluded patients with POD preoperatively, as they had received effective delirium management before surgery. Our prediction model aims to help clinicians identify high-risk patients for POD who may not have been recognized before surgery.

Our machine-learning models identified BNP, Troponin T, CRP, CK-MB, and other laboratory markers as the most important predictors of POD in hip fracture patients in the whole dataset. Intervening with these biomarkers may help reduce the incidence of POD in high-risk patients. In contrast, other machine-learning studies have identified well-known risk factors such as a history of stroke, preoperative delirium, preoperative dementia, preoperative mobility aid, and advanced age (older than 90) as important predictors of POD [16, 17]. These factors have been widely studied and cannot be modified [1, 2, 23, 26]. Therefore, our conclusion may have more practical implications for preventing POD in hip fracture patients by focusing on modifiable biomarkers that can be intervened upon to reduce the risk of POD. Besides, we introduce the SHAP to increase the interpretability of the model. The SHAP provides feature rankings for individual cases. It may help clinicians target specific interventions for patients at high risk of delirium, rather than employing a comprehensive approach for all patients. This individualized approach allows for a more efficient allocation of medical resources, as interventions can be tailored to address the specific contributing factors for each patient.

Despite its strengths, several limitations of our study should be acknowledged. First, it is a retrospective study. We used the DSM-IV criteria for POD by retrieving medical and nursing records [20]. Because the identification of POD based on the confusion assessment method (CAM) or 3D-CAM was not available in a retrospective study, this method may miss some hypoactive POD patients. Nevertheless, those with mixed and hyperactive POD patients always need urgent intervention for their poor prognosis [27]. The incidence of POD is 9.28%, which is lower, for we only identify the new-onset delirium after the surgery. Second, it is a single-center study, and only internal validation was performed. Therefore, extensive application of the model results may be limited. Third, although the AUC of our machine-learning model is acceptable compared with other machine-learning studies (AUC = 0.81) [16, 17], the performance of such machine-learning models can still be improved by exploring new algorithms.

In conclusion, we constructed six POD prediction models for patients with hip fractures using logistic regression, RF, AdaBoost, XGBoost, GBM, and SVM. The RF, one of five machine-learning modes, achieved the best AUC with 0.81. By providing convenient POD risk stratification, the application of machine-learning models can improve outcomes for elderly patients with hip fractures.

### Supplementary information

SUPPLEMENTAL MATERIAL

## Data Availability

The data that support the findings of this study are available from the corresponding author upon reasonable request.
