# Supplementary material for: Prediction models for postoperative delirium in elderly patients with machine-learning algorithms and SHapley Additive exPlanations
Source: Transl Psychiatry. 2024 Jan 25;14:57. doi: 10.1038/s41398-024-02762-w (PMC10808214; doi:10.1038/s41398-024-02762-w)
Supplement: Supplementary file 1 — SUPPLEMENTAL MATERIAL [file 41398_2024_2762_MOESM1_ESM.docx]

**Prediction models for postoperative delirium in elderly patients with machine-learning algorithms and SHapley Additive exPlanations**

**Yuxiang Song****^1†^, Di Zhang^1†^, Qian Wang^1†^, Yuqing Liu^1^, Kunsha Chen^1^, Jingjia Sun^1^, Likai Shi^1^, Baowei Li^1^, Xiaodong Yang^2^, Weidong Mi^1*^, Jiangbei Cao^1*^**

^1^ Department of Anesthesiology, The First Medical Center of PLA General Hospital, Beijing, China

^2^ Institute of Computing Technology, Chinese Academy of Sciences, Beijing, China

***Corresponding author:**

Jiangbei Cao, M.D., Ph.D. Professor of Anesthesiology, Department of Anesthesiology, The First Medical Center of PLA General Hospital, No.28, Fuxing road, Beijing, 100853, China. Email address:[caojiangbei@301hospital.com.cn](mailto:caojiangbei@301hospital.com.cn%20)

Wei-dong Mi, M.D., Ph.D. professor of Anesthesiology, Department of Anesthesiology, The First Medical Center of PLA General Hospital, No.28, Fuxing road, Beijing, 100853, China. Email: [wwdd1962@aliyun.com](mailto:wwdd1962@aliyun.com)

Yuxiang Song, Di Zhang, and Qian Wang contributed equally to the manuscript.

**†**These authors contributed equally to this work.

**Table S1 The univariate logistic regression analysis results of perioperative factors associated with POD.**

| **Variables** | Odds Ratio (95%CI) | P-value |
| --- | --- | --- |
| **Gender, male vs female** | 1.349(0.681-2.549) | 0.37 |
| **Age(years)** | 1.071(1.028-1.117) | 0.00115 |
| **BMI(kg·m-2)** | 0.955(0.886-1.027) | 0.221 |
| **Smoking, yes/quit smoking vs no** | 1.631(0.595-3.808) | 0.294 |
| **Drinking, yes/quit drinking vs no** | 1.028(0.24-3.028) | 0.965 |
| **Hypertension, yes vs no** | 0.708(0.39-1.285) | 0.254 |
| **Diabetes, yes vs no** | 1.793(0.974-3.261) | 0.0568 |
| **Coronary heart disease, yes vs no** | 0.984(0.415-2.072) | 0.968 |
| **Cerebrovascular disease, yes vs no** | 1.295(0.569-2.669) | 0.508 |
| **Depression/anxiety, yes vs no** |  |  |
| **Depression** | 2.469(0.369-9.947) | 0.257 |
| **Anxiety** | 23535333.082(0-NA) | 0.985 |
| **Renal insufficiency, yes vs no** | 4.111(1.28-11.327) | 0.00945 |
| **COPD, yes vs no** | 3.048(1.165-7.091) | 0.0142 |
| **Preoperative medication, yes vs no** |  |  |
| **Anticholinergics** | 0.776(0.123-2.705) | 0.735 |
| **Benzodiazepines** | 1.584(0.797-3.003) | 0.171 |
| **NSAIDs** | 1.332(0.071-7.489) | 0.789 |
| **Opioids** | 1.03(0.487-2.02) | 0.934 |
| **Antipsychotics** | 7.6(3.023-18.239) | <0.001 |
| **Troponin T(μg/L)** | 22.384(0.074-5991.327) | 0.191 |
| **Hemoglobin(g/L)** | 0.989(0.97-1.007) | 0.239 |
| **RBC count(*10^12^/L)** | 0.797(0.462-1.351) | 0.405 |
| **WBC count(*10^9^/L)** | 1.029(0.903-1.165) | 0.654 |
| **Neutrophils** | 49.407(2.182-1203.975) | 0.0153 |
| **Lymphocytes** | 0.006(0-0.349) | 0.0163 |
| **Monocytes** | 0.01(0-2603.286) | 0.482 |
| **Platelet count(*10^9^/L)** | 0.999(0.995-1.003) | 0.564 |
| **Glucose(mmol/L)** | 1.039(0.887-1.192) | 0.606 |
| **Serum albumin(g/L)** | 0.932(0.858-1.008) | 0.087 |
| **Myoglobin quantification(ug/L)** | 1.005(1.001-1.009) | 0.0117 |
| **BUN(mmol/L)** | 1.047(0.968-1.118) | 0.201 |
| **Scr(μmol/L)** | 1.003(0.998-1.007) | 0.181 |
| **Serum uric acid(****μmol/L)** | 0.998(0.994-1.001) | 0.258 |
| **Serum potassium(mmol/L)** | 1.4(0.635-2.984) | 0.393 |
| **Serum sodium(mmol/L)** | 1.017(0.943-1.102) | 0.676 |
| **BNP(pg/mL)** | 1(1-1) | 0.0699 |
| **Serum calcium(mmol/L)** | 1.095(0.113-9.455) | 0.936 |
| **Chloride(mmol/L)** | 1.048(0.977-1.127) | 0.1973 |
| **Inorganic phosphorus(mmol/L)** | 0.211(0.05-0.862) | 0.0321 |
| **Magnesium(mmol/L)** | 0.699(0.018-28.249) | 0.849 |
| **Total bilirubin(μmol/L)** | 1.029(0.995-1.061) | 0.0713 |
| **Direct bilirubin(μmol/L)** | 1.103(1.011-1.196) | 0.019 |
| **ALT(U/L)** | 1.006(0.987-1.022) | 0.483 |
| **AST(U/L)** | 1.019(0.999-1.037) | 0.0421 |
| **LDH(U/L)** | 1.002(0.998-1.006) | 0.179 |
| **CK(U/L)** | 1.001(0.998-1.004) | 0.341 |
| **CK-MB(U/L)** | 1.142(0.947-1.351) | 0.116 |
| **GGT(U/L)** | 1.006(0.997-1.014) | 0.151 |
| **ALP(U/L)** | 1.003(0.992-1.011) | 0.59 |
| **Amylase(U/L)** | 0.985(0.97-0.999) | 0.0543 |
| **Lipase(U/L)** | 0.994(0.987-0.999) | 0.041 |
| **CRP(mg/L)** | 1.091(1.009-1.174) | 0.0211 |
| **PO_2_(mmHg)** | 0.992(0.975-1.008) | 0.371 |
| **PCO_2_(mmHg)** | 0.965(0.913-1.017) | 0.189 |
| **SPO_2_(%)** | 0.884(0.814-0.957) | 0.00241 |
| **HCO_3_(mmol/L)** | 0.97(0.865-1.087) | 0.606 |
| **BE(mmol/L)** | 1.006(0.889-1.139) | 0.923 |
| **TT(s)** | 1.019(0.853-1.163) | 0.8072 |
| **APTT(s)** | 1.011(0.964-1.057) | 0.63926 |
| **PT(s)** | 1.213(1.018-1.424) | 0.0209 |
| **PTA(%)** | 0.973(0.953-0.993) | 0.00818 |
| **INR** | 7.987(1.445-39.573) | 0.0117 |
| **FIB(g/L)** | 0.925(0.703-1.205) | 0.56957 |
| **Plasma D dimer(mg/L)** | 0.981(0.863-1.09) | 0.75 |
| **Type of hip fracture vs Femoral neck fracture** |  |  |
| **Intertrochanteric fracture** | 1.127(0.622-2.049) | 0.693 |
| **Others** | 0(NA-9.64604937158001e+24) | 0.987 |
| **Type of surgery vs Closed reduction and internal fixation** |  |  |
| **Open reduction and internal fixation** | 1.049(0.579-1.907) | 0.875 |
| **Joint replacement surgery** | 0(NA-9.30129034916746e+24) | 0.987 |
| **Duration of operation(min)** | 0.993(0.984-1.002) | 0.16 |
| **Duration of anesthesia(min)** | 0.996(0.987-1.004) | 0.317 |
| **ASA, vs I** |  |  |
| **II** |  | 0.993 |
| **III** |  | 0.993 |
| **IV** |  | 0.992 |
| **V** |  | 0.988 |
| **Type of Anesthesia, vs Spinal anesthesia** |  |  |
| **Nerve block** | 4.765(0.862-89.022) | 0.1435 |
| **General anesthesia** | 3.818(0.655-72.343) | 0.2157 |
| **General anesthesia combined with other modalities** | 6.591(1.368-118.563) | 0.0663 |
| **Urine(ml)** | 0.999(0.997-1) | 0.142 |
| **Blood loss(ml)** | 1(0.997-1.002) | 0.693 |
| **Colloid(ml)** | 1(0.999-1.001) | 0.791 |
| **Crystal(ml)** | 1(0.999-1.001) | 0.496 |
| **Blood transfusion, yes vs no** | 1.787(0.98-3.245) | 0.056 |
| **Intraoperative medication, yes vs no** |  |  |
| **Glucocorticoids** | 1.23(0.648-2.261) | 0.514 |
| **Dexmedetomidine** | 1.347(0.667-2.576) | 0.384 |
| **Droperidol** | 1.409(0.556-3.116) | 0.428 |
| **Vasoactive drugs** | 2.3(1.201-4.278) | 0.00971 |
| **Intraoperative blood pressure** |  |  |
| **Duration of SBP>=140mmHg (min)** | 1.001(0.992-1.009) | 0.906 |
| **Duration of MAP<=60mmHg(min)** | 1.013(0.987-1.036) | 0.286 |
| **Preoperative hospital stay (days)** | 1.073(0.987-1.16) | 0.0855 |

**Table S2. The exact data of importance of all the variables.**

| **Variables** | **Importance** | **Normalized importance** |
| --- | --- | --- |
| **BNP** | 54 | 0.077021823 |
| **Troponin T** | 27.6 | 0.039366709 |
| **CRP** | 24.7 | 0.035230352 |
| **CK-MB** | 22.9 | 0.032662958 |
| **SPO_2_** | 21.9 | 0.031236628 |
| **GGT** | 21.7 | 0.030951362 |
| **PaO_2_** | 21.2 | 0.030238197 |
| **Serum Calcium** | 20.9 | 0.029810298 |
| **CK** | 19.3 | 0.02752817 |
| **Chloride** | 17.7 | 0.025246042 |
| **Age** | 17.6 | 0.025103409 |
| **Amylase** | 16.3 | 0.02324918 |
| **Myoglobin quantification** | 15.7 | 0.022393382 |
| **BE** | 12.9 | 0.018399658 |
| **Intraoperative maximum systolic blood pressure** | 12 | 0.017115961 |
| **ALT** | 12 | 0.017115961 |
| **Red blood cell count** | 11.7 | 0.016688062 |
| **Serum potassium** | 11.6 | 0.016545429 |
| **LDH** | 11.3 | 0.01611753 |
| **Serum uric acid** | 11.3 | 0.01611753 |
| **Plasma D-dimer** | 11 | 0.015689631 |
| **ALP** | 10.9 | 0.015546998 |
| **Neutrophils** | 10.9 | 0.015546998 |
| **APTT** | 10.8 | 0.015404365 |
| **PaCO_2_** | 10.4 | 0.014833833 |
| **Preoperative hospital stay** | 10.4 | 0.014833833 |
| **White blood cell count** | 10.4 | 0.014833833 |
| **Intraoperative - Dexmedetomidine** | 9.8 | 0.013978035 |
| **Glucose** | 9.8 | 0.013978035 |
| **Platelet count** | 9.5 | 0.013550136 |
| **CO_2_** | 9.2 | 0.013122236 |
| **Lipase** | 8.8 | 0.012551704 |
| **AST** | 8.6 | 0.012266438 |
| **Direct bilirubin** | 8.5 | 0.012123805 |
| **BUN** | 8.3 | 0.011838539 |
| **Plasma fibrinogen** | 8.3 | 0.011838539 |
| **BMI** | 8.2 | 0.011695906 |
| **Serum albumin** | 8 | 0.01141064 |
| **TT** | 7.9 | 0.011268007 |
| **Antipsychotics** | 7.7 | 0.010982741 |
| **Benzodiazepines** | 7.4 | 0.010554842 |
| **Intraoperative maximum MAP** | 7.2 | 0.010269576 |
| **Serum sodium** | 7.1 | 0.010126943 |
| **Hemoglobin** | 7 | 0.00998431 |
| **HCO_3_** | 7 | 0.00998431 |
| **PT** | 6.8 | 0.009699044 |
| **Scr** | 6.7 | 0.009556411 |
| **Intraoperative maximum diastolic blood pressure** | 6.6 | 0.009413778 |
| **Time for intraoperative systolic blood pressure>=140(min)** | 5.7 | 0.008130081 |
| **Inorganic Phosphorus** | 5.6 | 0.007987448 |
| **Duration of anesthesia** | 5.1 | 0.007274283 |
| **Prothrombin activity** | 4.8 | 0.006846384 |
| **Crystal** | 4.7 | 0.006703751 |
| **Duration of Operation** | 4.7 | 0.006703751 |
| **Total bilirubin** | 4.1 | 0.005847953 |
| **Lymphocytes** | 3.9 | 0.005562687 |
| **INR** | 3.9 | 0.005562687 |
| **Magnesium** | 3.6 | 0.005134788 |
| **Colloid** | 2.9 | 0.004136357 |
| **Cerebrovascular disease** | 2.9 | 0.004136357 |
| **ASA** | 2.6 | 0.003708458 |
| **Urine output** | 2.5 | 0.003565825 |
| **Monocytes** | 2.3 | 0.003280559 |
| **Amount of bleeding** | 2 | 0.00285266 |
| **Blood transfusion or not** | 1.8 | 0.002567394 |
| **The duration of intraoperative MAP<60** | 1.8 | 0.002567394 |
| **Anesthesia classification** | 1.2 | 0.001711596 |
| **Sex** | 1.1 | 0.001568963 |
| **COPD** | 1 | 0.00142633 |
| **Intraoperative vasoactive drugs** | 0.9 | 0.001283697 |
| **Hypertension** | 0.8 | 0.001141064 |
| **Time for intraoperative diastolic blood pressure>=90 (min)** | 0.6 | 0.000855798 |
| **Intraoperative - droperidol** | 0.5 | 0.000713165 |
| **Diabetes** | 0.5 | 0.000713165 |
| **Type of hip fracture** | 0.5 | 0.000713165 |
| **Type of surgery** | 0.4 | 0.000570532 |
| **Intraoperative glucocorticoids** | 0.3 | 0.000427899 |
| **Opioids** | 0.3 | 0.000427899 |
| **Renal insufficiency** | 0.2 | 0.000285266 |
| **Coronary heart disease** | 0.2 | 0.000285266 |
| **Intraoperative systolic blood pressure>=140** | 0.1 | 0.000142633 |
| **Smoking** | 0.1 | 0.000142633 |
| **Depression or anxiety** | 0 | 0 |
| **Drinking** | 0 | 0 |
| **Anticholinergics** | 0 | 0 |
| **NSAIDs** | 0 | 0 |


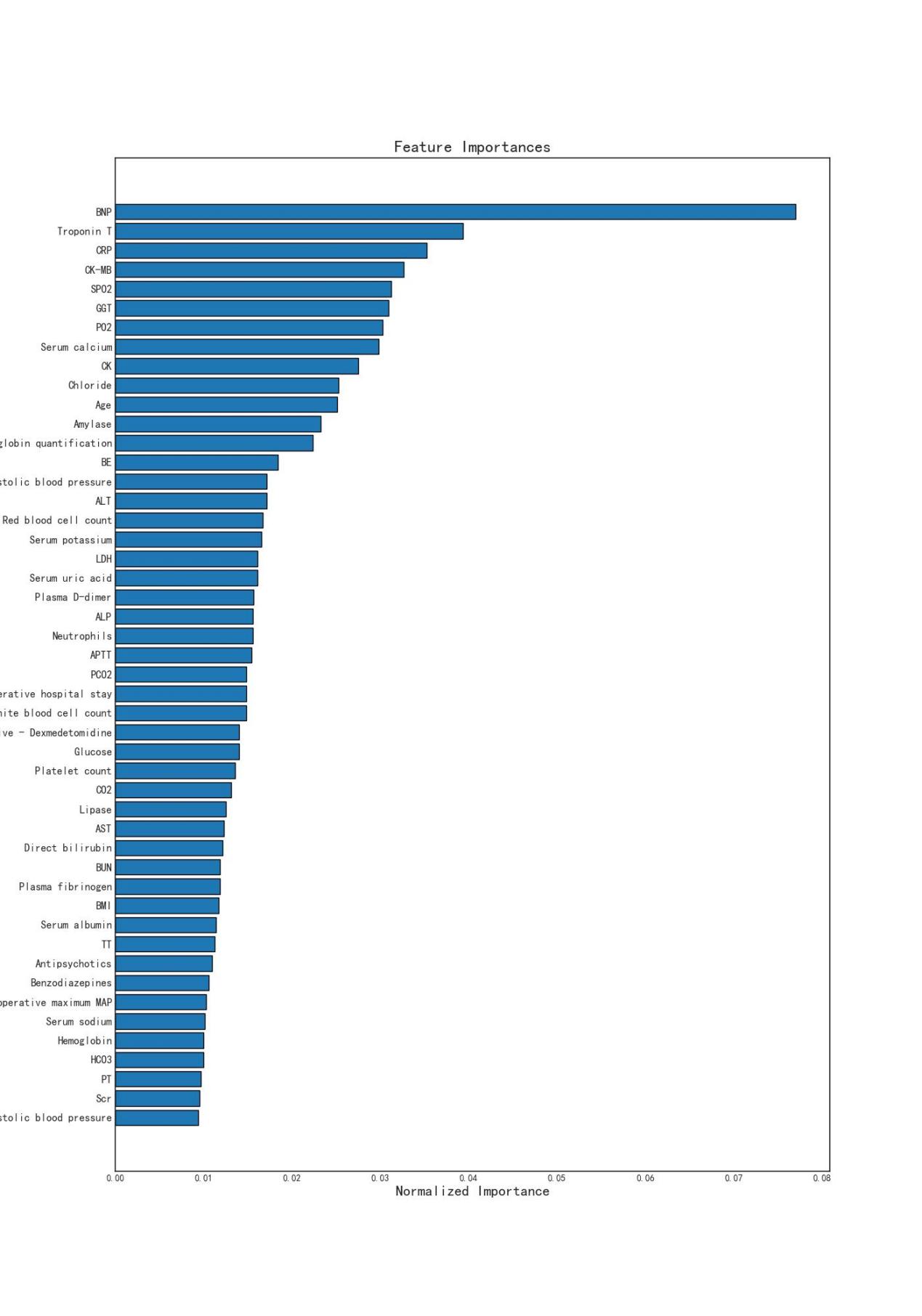


**Figure S1 The importance of all the variables.**

**
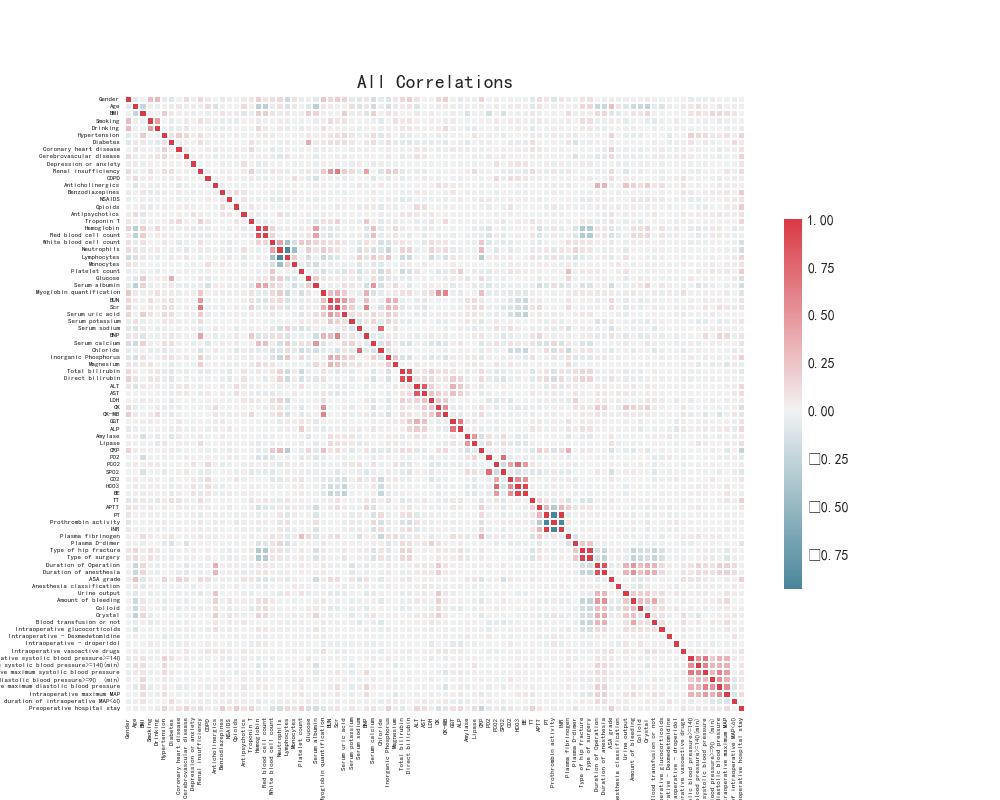
**

**Figure S2 The heatmap of all the variables correlation.**
